# Development of Promising Interventions to Improve Human Papillomavirus Vaccination in a School-Based Program in Quebec, Canada: Results From a Formative Evaluation Using a Mixed Methods Design

**DOI:** 10.2196/57118

**Published:** 2024-07-08

**Authors:** Maude Dionne, Chantal Sauvageau, Doriane Etienne, Marilou Kiely, Holly Witteman, Eve Dubé

**Affiliations:** 1 Institut National de Santé Publique du Québec Quebec, QC Canada; 2 Infectious and Immune Diseases Axis Centre de Recherche du CHU de Québec-Université Laval Québec, QC Canada; 3 Faculty of Medicine Université Laval Québec, QC Canada; 4 VITAM—Centre de Recherche en Santé Durable Québec, QC Canada; 5 Population Health and Optimal Health Practices Axis Centre de Recherche du CHU de Québec-Université Laval Québec, QC Canada; 6 Faculty of Social Sciences Université Laval Québec City, QC Canada

**Keywords:** immunization, human papillomavirus, HPV, HPV vaccine, school-based immunization program, intervention, strategies, vaccination, vaccine, Quebec, school-based, vaccine coverage, decision aid, student, students, nurse, nurses, parent, parents, focus group, descriptive analyses, user-centered, effectiveness, data collection, vaccine safety

## Abstract

**Background:**

Despite the availability of school-based human papillomavirus (HPV) vaccination programs, disparities in vaccine coverage persist. Barriers to HPV vaccine acceptance and uptake include parental attitudes, knowledge, beliefs, and system-level barriers. A total of 3 interventions were developed to address these barriers: an in-person presentation by school nurses, an email reminder with a web-based information and decision aid tool, and a telephone reminder using motivational interviewing (MI) techniques.

**Objective:**

Here we report on the development and formative evaluation of interventions to improve HPV vaccine acceptance and uptake among grade 4 students’ parents in Quebec, Canada.

**Methods:**

In the summer of 2019, we conducted a formative evaluation of the interventions to assess the interventions’ relevance, content, and format and to identify any unmet needs. We conducted 3 focus group discussions with parents of grade 3 students and nurses. Interviews were recorded, transcribed, and analyzed for thematic content using NVivo software (Lumivero). Nurses received training on MI techniques and we evaluated the effect on nurses’ knowledge and skills using a pre-post questionnaire. Descriptive quantitative analyses were carried out on data from questionnaires relating to the training. Comparisons were made using the proportions of the results. Finally, we developed a patient decision aid using an iterative, user-centered design process. The iterative refinement process involved feedback from parents, nurses, and experts to ensure the tool’s relevance and effectiveness. The evaluation protocol and data collection tools were approved by the CHU (Centre Hospitalier Universitaire) de Québec Research Ethics Committee (MP-20-2019-4655, May 16, 2019).

**Results:**

The data collection was conducted from April 2019 to March 2021. Following feedback (n=28) from the 3 focus group discussions in June 2019, several changes were made to the in-person presentation intervention. Experts (n=27) and school nurses (n=29) recruited for the project appreciated the visual and simplified information on vaccination in it. The results of the MI training for school nurses conducted in August 2019 demonstrated an increase in the skills and knowledge of nurses (n=29). School nurses who took the web-based course (n=24) filled out a pretest and posttest questionnaire to evaluate their learning. The rating increased by 19% between the pretest and posttest questionnaires. Several changes were made between the first draft of the web-based decision-aid tool and the final version during the summer of 2019 after an expert consultation of experts (n=3), focus group participants (n=28), and parents in the iterative process (n=5). More information about HPV and vaccines was added, and users could click if more detail is desired.

**Conclusions:**

We developed and pilot-tested 3 interventions using an iterative process. The interventions were perceived as potentially effective to increase parents’ knowledge and positive attitudes toward HPV vaccination, and ultimately, vaccine acceptance. Future research will assess the effectiveness of these interventions on a larger scale.

## Introduction

Human papillomavirus (HPV) is one of the most commonly transmitted sexual infections [1]. There are over 200 types of HPV, some of which cause cancer (eg, cervical, oropharyngeal, and genital) or genital warts (ie, condylomas) [1]. Most of the oncogenic HPV types can be prevented by vaccination [1]. In Quebec, Canada, HPV vaccination has been offered as part of school-based vaccination programs to boys and girls in grade 4 since 2008. Despite school-based programs providing equitable access to vaccination, multiple studies have shown that there are still major disparities in vaccine coverage and that students who live in socially and materially deprived areas and areas with a higher proportion of immigrants have lower HPV vaccine coverage [2,3].

In Quebec, Canada, the HPV vaccination routine program has been offered to girls in grade 4 since 2008 [4]. The vaccine coverage for 2 doses was 81% for the first year (2008-2009) [5]. During the subsequent years, a decrease was observed as the vaccine uptake coverage fell to 73% in 2015-2016 (2 doses) [6]. The drop in HPV vaccination coverage could be explained by the usual increase observed after the first few years of implementing a new publicly funded vaccination program. The school HPV vaccination program was the subject of criticism and concern by the public, which may have had a negative effect on uptake and explained the fluctuation in vaccine coverage over the following years [7-9]. Boys were integrated into the program in September 2016. In 2017-2018, the uptake coverage (2-dose) increased to 78% for girls and 75% for boys [10], and it was still slightly higher for girls than for boys in 2021-2022 (83% vs 79%) [11].

Multiple factors could explain a lower vaccine coverage in boys, such as sociodemographic factors, belief-related variables, family factors, community factors, and needs and environmental factors, as stated in a systematic review published in 2022 [12]. Furthermore, as vaccination of boys only began in 2016, they had fewer opportunities than girls to be vaccinated. Various projects have been carried out in Quebec to explore the barriers to HPV vaccination in school vaccination programs that the vaccine coverage did not reach. These projects identified key barriers to HPV vaccine acceptance and uptake that were related to both parental attitudes, knowledge, and beliefs (eg, fear of long-term side effects such as the onset of chronic diseases, low perception of the benefits of vaccines) and system-level barriers (eg, informed consent process, communication, and promotion tools) [8,13,14]. A key barrier remains parents’ missing informed consent forms due to lack of time, lack of collaboration between schools and public health, or miscommunications. Various strategies and actions were proposed by the key informants consulted. These included improving the information provided to parents (eg, parent information sessions) and better support to school nurses (ie, training and availability of tools) [8].

Although there is strong evidence that different interventions are effective in enhancing vaccine acceptance and uptake, fewer studies have tested interventions targeting specifically HPV vaccination in school-based programs [15,16]. Reminder and recall interventions (by mail, telephone, or text) effectively increase vaccine coverage [16-24]. A study showed that information and education activities targeting parents and health care workers to raise awareness and knowledge about HPV infection and vaccines improved vaccine acceptance [20]. Furthermore, web-based interventions (social media and websites) with vaccine information for parents also positively impacted HPV vaccine uptake [16] or willingness to consider the HPV vaccine for their children [25].

Multiple studies and systematic reviews concluded that a recommendation from health care workers was an influential factor in enhancing HPV vaccine acceptance [18-20,26]. For example, a randomized controlled trial for an HPV vaccine communication intervention with parents concluded that receipt of a strong recommendation was associated with greater perceived urgency to vaccinate, greater confidence in the information provided by the provider, decreased hesitancy to vaccinate, and increased vaccine administration [27,28]. A study conducted in the United States also showed that perceived parental HPV vaccine hesitancy was significantly associated with provider-level factors such as self-efficacy, outcome expectations, and confidence in HPV vaccine safety [29].

In addition to equipping health professionals to communicate with parents about HPV vaccines, providing parents with validated, detailed information sources they can consult outside of clinical encounters may be helpful. One way to structure such information is the form of a patient decision aid. Patient decision aids are tools, such as pamphlets or websites, explaining the benefits and harms of different health options. Unlike more traditional public health or medical information sources, patient decision aids explicitly support human decisional processes by making the decision explicit, providing balanced information [30] on potential benefits and harms of options [31,32], and—similar to the way motivational interviewing (MI) draws on personal values—helping people clarify what matters to them relevant to the decision [33]. The Cochrane Review of patient decision aids for screening and treatment decisions showed that these tools help people make better-informed health decisions and feel better about their decisions [34]. A systematic review and meta-analysis of 5 patient decision aid for vaccine decisions (n=2158) addressing a range of vaccines (measles, mumps, and rubella; influenza; diphtheria, tetanus, pertussis, and *Haemophilus influenzae* type B; polio; and hepatitis B) showed that the overall effect estimate of patient decision aids was 1.89 (95% CI 1.20-2.97) on vaccine intentions and 1.77 (95% CI 1.25-2.52) on vaccine uptake [35].

In this context, a qualitative and exploratory project was carried out to develop interventions to improve the HPV vaccine acceptance and vaccine coverage in Quebec’s school-based vaccination program. The Ministère de la Santé et des Services sociaux and regional health authorities of selected regions closely collaborated in the design of the project and the intervention development and evaluation. The first step of the project aimed to develop 3 interventions based on data from the literature and consultation with key stakeholders involved in school vaccination delivery (Textbox 1). This paper presents the results of the intervention development process and formative evaluation of those interventions.

**The 3 interventions description.**

**Intervention 1: in-person presentation**

- An in-person presentation by school nurses to parents of 4th-grade students at the beginning of the school year to discuss the vaccines offered to students. This was supported by an evidence-based visual presentation.

**Intervention 2: email reminder using a web-based information and decision aid tool**

- An email reminder was sent to parents who had not returned the consent form before vaccination, along with a web link to a web-based information and decision aid tool. This educational tool provided neutral, evidence-based information in a visual format accessible to all literacy levels and allophones.

**Intervention 3: telephone reminder using an approach inspired by motivational interviewing (MI) techniques**

- Parents who had not returned the consent form following the email reminder (intervention 2) received a telephone reminder from the school nurse trained in MI. MI is a brief intervention style based on empathic listening without argumentation and respecting the parent’s autonomy. It helps an individual make an informed decision, reinforcing motivation and commitment to health-promoting behavior [36].

As noted previously, in Quebec, the HPV immunization program was introduced in 2008, first targeting girls in grade 4 of primary school, with boys included since 2016. The vaccine coverage varies from region to region and overall falls short of the 90% target for optimal cervical cancer prevention [25,26,37].

The delivery of HPV vaccines in schools varies slightly between areas and schools [8]. Generally, school vaccination is carried out in masse over a short period (eg, one full day in a school). Most of the time, public health school nurses in charge of a school will visit classes in the weeks before the vaccination to talk to students about vaccination and give them the information brochure, including the consent form, to be signed by parents to accept or decline the vaccination [21]. In the days before vaccination, reminders may be sent to students who have not returned the consent form. Sometimes, a letter is sent to the parent, or the school nurse makes a call.

School nurses are involved in health promotion, prevention, and protection, as well as health maintenance and restoration in schools [38]. They are employed by local health authorities. Most of the time, vaccination is offered by local health authorities to apply the provincial program of the Ministère de la Santé et des Services sociaux [38]. School nurses carry out vaccination activities of the program (eg, vaccination schedule, information, and consent) to youths in local schools where they are working. They organize vaccination sessions in collaboration with the school administration. As a vaccinator, the nurse must promote and recommend vaccination after explaining the risks and benefits to parents or youths of age to consent [38]. They are responsible for verifying the consent form before vaccinating. Nurses often have to deal with challenges such as a limited time of presence in each school, a large number of students and schools, and a vast territory to cover [38]. As an indication, the school nurses who took part in this project in 2019-2020 had an average of 6 schools under their responsibility, with this number varying from 2 to 18 schools.

## Methods

### Overview

A formative evaluation of the interventions was carried out in the summer of 2019 to assess the interventions’ relevance, content and format, and any missing elements (unmet needs; Table 1). Formative evaluation is “a rigorous assessment process designed to identify potential and actual influences on the progress and effectiveness of implementation efforts” [39]. Data from the formative evaluation are shared with the implementation team to adapt and improve the implementation process of interventions during the course of the project presented in this paper [40].

**Table 1:** Interventions’ development and formative evaluation process from March to August 2019.

Tasks			March		April		May		June		July		August
**Intervention development**													
	Consulting stakeholders and experts	✓		✓		✓							
	Develop and validate interventions’ content	✓		✓		✓						✓	
**Recruitment**													
	Schools and nurses’ recruitment							✓		✓		✓	
**Formative evaluation**													
	Focus group with parents of grade 3 students and school nurses							✓					
**Final version**													
	Motivational interview training									✓		✓	
	Patient decision aid iterative development									✓		✓	
	Final version of interventions (in-person meeting presentation and decision-aid)											✓	

A consultation with experts in intervention methods, vaccination, and HPV was done to assess the content and format of the information presented.

### Recruitment of Schools

The proposed method is based on feasibility factors and is inspired by the Matusita technique [41]. Pairs of sociodemographically comparable schools with low HPV vaccine coverage in the 3 regions of Quebec with the lowest HPV vaccine coverage (Montreal, Laval, and Laurentides) were selected.

To detect a 10% increase in HPV vaccine coverage between schools in the experimental and control groups, assuming a vaccine coverage baseline of 50%, power of 80%, α risk of 5%, and intraschool correlation of 0.05, we needed to recruit 32 schools (approximately 11 schools or regions) in the experimental group and 32 schools in the control group. There were 64 schools selected for the project based on certain criteria (eg, HPV vaccine coverage, number of students, deprivation index). Then, 2 groups of similar schools were created for comparison, half of which were targeted to pilot-test the interventions, and the other half was used as a control with vaccination activities delivered as usual (ie, vaccine presentation to students, a reminder to parents for returning the consent form not using a decision-air tool or MI technics). The final selection and assignment were made in consultation with immunization officers, school nurses, and school principals, considering feasibility factors (ie, availability of nurses, the willingness of school principals, and favorable school context).

### Nurses’ Presentations at School-Based Parent Meetings

An external research firm was in charge of recruiting and organizing focus groups in the Quebec City area in June 2019. Participants had to complete a short recruitment questionnaire to be invited to the group discussion (Multimedia Appendix 1). Two groups of parents of grade 3 students and 1 group of school nurses were recruited. We targeted parents of grade 3 children, as they had not yet been informed of the vaccination offered in grade 4 by the school nurse nor received the brochure from the Ministère de la Santé et des Services sociaux. As a result, they were in a better position to assess the relevance of the content of the interventions and determine whether it enabled them to fully understand HPV and the vaccination offered. Participants viewed and commented on the presentation’s content for the exchange meeting between school nurses and parents of grade 4 students (intervention 1) and the content of the information and decision aid tool developed by a team from Université Laval (intervention 2).

Focus groups were structured to present the content of the presentation for the nurses’ meeting and the decision aid tool. An interview guide was designed to assess focus group participants’ perception of the interventions’ content. Questions about their first impressions, credibility, trust in information, satisfaction with information, clarity of information, and possible improvements were asked. Focus groups and individual interviews were recorded and transcribed. The transcripts were analyzed for thematic content using NVivo software (Lumivero) [42]. The data were categorized into categories and subcategories that followed the themes discussed during the interviews with the questionnaire.

### MI Training of School Nurses

Each school nurse in charge of the pilot schools was recruited (n=25). In line with intervention 3, these school nurses assigned to the pilot schools recruited were trained in MI techniques during the summer of 2019. The training took place in several stages. An initial 5-hour web-based training course provided a theoretical basis [43] on the MI style, spirit, the 4 processes, essential skills, and processes in action. A pretest and posttest measured various skills and knowledge such as resistance and ambivalence, the MI process, interpersonal skills, types of discourse, and practice using clinical vignettes. Nurses obtained a rating filling those tests. The test had 40 questions, and it took around 15 minutes to complete. The rating is a total of 40 points (1 point for each correct answer). After that, a 1-day face-to-face training session with an expert consolidated learning through practice. An integration workshop was also organized to give the school nurses practice with a fictitious call to a hesitant parent. Finally, the qualified trainer gave each nurse individual personalized feedback after these calls.

Descriptive quantitative analyses were carried out on data from questionnaires relating to MI training. Pre- and postcomparisons of obtained ratings (on 40 points) were made.

### Patient Decision Aid Formative Development

**Figure 1:**
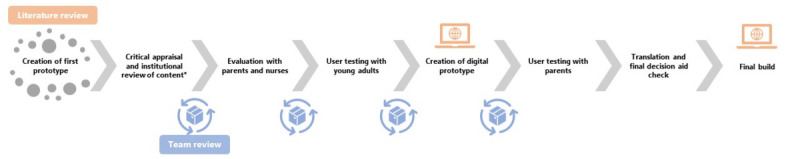
Iterative development cycles of the decision aid tool based on user-centered design.

In alignment with international guidelines about patient decision-aid development [44], an iterative, user-centered design approach was used to develop a web-based patient decision-aid about HPV vaccination (Figure 1). First, a multidisciplinary team (3 people) with expertise in vaccine hesitancy, communications, user experience design, and epidemiology collaborated to draft an initial prototype of the patient decision aid. Second, content experts (9 experts from advisory committee and 18 experts from coordinating committee) at the provincial public health agency and its health system partners conducted a critical appraisal and review of the content during the summer of 2019. Third, participants in the focus groups (N=28 participants; Table 2 ) were invited to comment on the evolving prototype patient decision aid and other interventions. Fourth, female Cégep (precollege) students (n=3) known to the research team were invited to comment on the prototype patient decision aid and provide perspectives as people directly impacted by HPV vaccination decisions. The fifth iterative cycle unfolded in 2 phases. In the first phase, a nuanced, page-by-page analysis was conducted by 2 parents of children eligible for HPV vaccines (n=2) in a cognitive interview setting, evaluating a paper prototype of the patient decision aid. In the second phase, 3 parents of children eligible for HPV vaccines (n=3) participated in user testing sessions of the digital prototype of the patient decision aid, following a think-aloud protocol. Sixth and finally, the final version of the patient decision aid was translated from French to English and validated by the original multidisciplinary experts (n=3). Throughout this iterative process, experts were recruited via personal contacts. Parents who participated in user testing were recruited by posting open recruitment messages on university-based electronic mailing lists (listserves). The critical appraisal and review of content were conducted by the Institut National de Santé Publique du Québec (INSPQ) and its institutional partners from the health and social service network.

**Table 2:** Characteristics of focus group participants.

Characteristics		Parents of grade 3 children (n=10), n (%)	Parents of grade 3 children (n=10), n (%)	School nurses (n=8), n (%)
**Sex**				
	Women	7 (70)	5 (50)	7 (88)
	Men	3 (30)	5 (50)	1 (12)
**Children’s sex**				
	Girls	5 (50)	6 (60)	—^a^
	Boys	5 (50)	4 (40)	—^a^
**Age (years)**				
	25-34	2 (20)	0	—^a^
	35-44	6 (60)	8 (80)	—^a^
	≥45	2 (20)	2 (20)	—^a^
**Education level**				
	High school	0	1 (10)	—^a^
	College	3 (30)	6 (60)	—^a^
	University	7 (70)	3 (30)	—^a^

^a^Not available.

Comments on the different versions of the prototype patient decision aid and observations from user testing were compiled into lists of potential issues to address. The patient decision aid development team then analyzed each list and assessed each issue’s severity and frequency. In other words, would the issue cause major problems to users, such as misinterpretations of content or inability to proceed through the patient decision aid, and the frequency of these problems (frequent or rare). The team then prioritized changes for the next version, and the prototype patient decision aid was changed in response.

### Ethical Considerations

The evaluation protocol and data collection tools were approved by the CHU (Centre Hospitalier Universitaire) de Québec Research Ethics Committee (project number: MP-20-2019-4655, approved on May 16, 2019). Schools’ principals were contacted by email and by phone by a member of the research team to discuss the project and consent to participate (written or verbal consent). Transcripts were created from focus group recordings and deidentified before analysis. Informed consent was obtained from all focus group participants recruited to the project.

Ethical approval for developing a patient decision aid for the HPV vaccine and its complementary video was obtained from the Health Research Ethics Committee of Université Laval (2019-106/24-05-2019). All participants provided written informed consent before enrollment. Participants were compensated for their time.

## Results

### Nurses’ Presentations at School-Based Parent Meetings

#### Focus Group

A total of 3 groups were conducted, 2 with parents and 1 with school nurses. The characteristics of participants in the 3 groups are shown in Table 2. All participating parents generally had a positive attitude about vaccination (15/20, 75%) and accepted all vaccines, and 5 (25%) accepted some vaccines. A total of 15 (75%) participants had vaccinated their child with all the recommended vaccines, and 5 (25%) had postponed or refused certain vaccines.

#### Results of a Focus Group With Parents

After reviewing the slides for the planned nurses’ presentations at parent-school meetings, parents wanted to know more concretely what HPVs are and what diseases they cause. A total of 4 parents found it too long (33 PowerPoint slides), while another felt the content was too general. For 2 parents, the content was reassuring. A total of 2 parents wanted more information on side effects (eg, long-term potential side effects and duration of possible side effects): “I want to know about side effects other than those caused by vaccines in general. The risks my child is exposed to with the HPV vaccine.” One parent felt there was too much emphasis on sexual relations: “They talk a lot about sexual relations, but they don’t talk about the problems that can arise when on trips, for example. I don’t know if it concerns 8-year-olds that much. That doesn’t convince me enough.” One parent suggested explaining the importance of vaccination at the outset. A total of 4 parents wanted sources of data and evidence on the safety of vaccines to be added to give them confidence. To answer their questions, the following suggestions for additions were collected: the vaccination schedule, vaccine ingredients, contact details for vaccination centers, possible side effects, duration of efficacy, and the vaccination process (eg, immunization schedule, receiving 2 vaccines on the same day, HPV and Hepatitis B). Some parents preferred data with statistics (eg, efficiency percentage) and data from Quebec. They wanted more information on vaccine efficacy for both boys and girls. The majority appreciated the presentation of data in a simple, schematic form.

**Figure 2:**
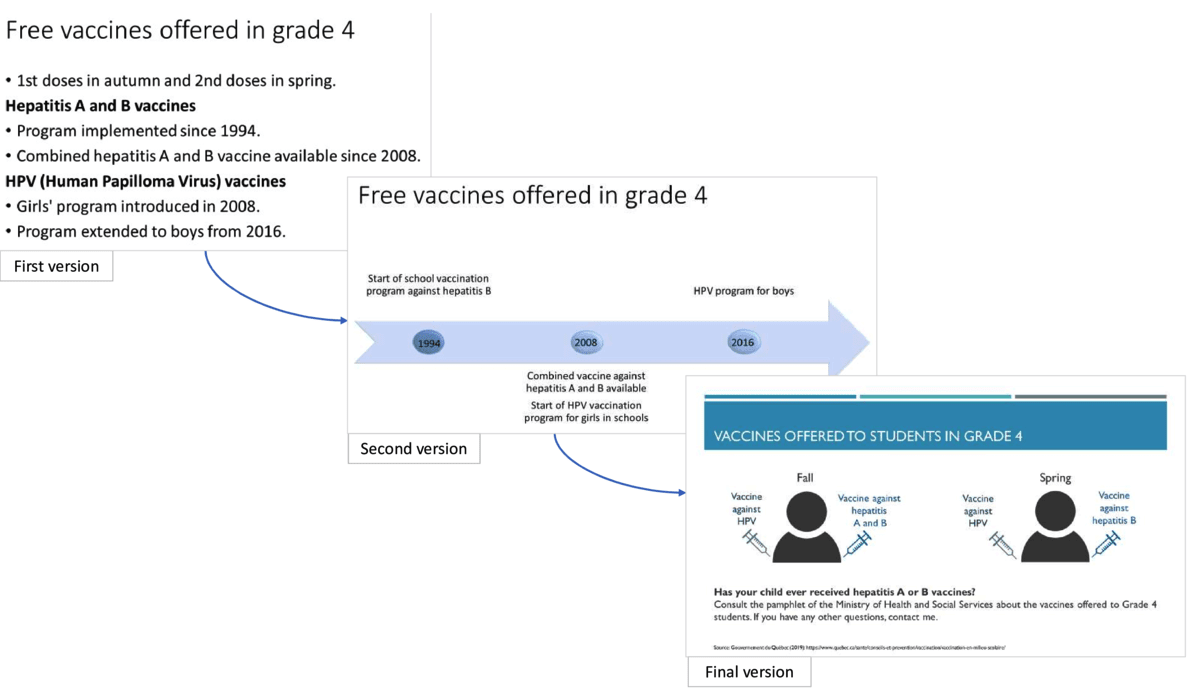
Example of changes in intervention 1.

Following feedback from the parent’s focus groups, several changes were made to the presentation (intervention 1: in-person presentation by school nurses). First, the presentation was significantly shortened from 33 to 12 slides, giving a presentation of no more than 10 minutes. This short presentation was perceived as simplified, while only the essential information requested by the parents and nurses was preserved. Information on the vaccination calendar (eg, which vaccines are offered and when), the importance of vaccinating boys (eg, transmission, screening, and associated cancers), the ideal age (eg, more effective before sexual relations and good immune response), and vaccine efficacy (eg, Quebec’s data) and safety (eg, possible side effects and safety monitoring) were added, as well as more detailed statistics and data sources. The final version covered the following topics: the vaccination schedule, HPV in brief, the course of HPV infection, HPV-related cancers, vaccine efficacy, possible side effects, vaccine safety, and additional resources. The visual format of the information was also simplified and illustrated (Figure 2).

The modified version was presented to parents in the second group. Feedback received indicated that many parents felt there was too much information on preparing the child and not enough on the potential risk of side effects after HPV vaccination. This group also suggested adding more sources, statistics, and images. Information on HPV, vaccination, boys, and side effects was also requested: “We talk a lot about girls and screening. But for guys, we don’t even know how they know whether they're infected.” The majority agreed that the length of the presentation was sufficient. In general, parents were more appreciative of the new version of intervention 1.

#### Results of the Focus Group With Nurses

The nurses felt that the content was relevant and presented the same information as the information brochure from the Ministère de la Santé et des Services sociaux given to parents with the consent form [45]. It was suggested that certain information be clarified, notably on vaccine efficacy, child preparation, and the importance of vaccination in infancy. They recommended keeping the information simple, transparent, and precise to do this. To this end, some appreciated the use of images to help clarify information. They felt it was important to reassure parents about possible side effects and to take the opportunity to deconstruct certain myths that persist: “Saying that vaccines don’t cause illness. It’s like people are afraid it will give them the disease.”

As for the feasibility of carrying out the intervention, some nurses voiced concerns such as the difficulty of mobilizing parents, attracting those who refuse vaccination, and keeping their attention during the meeting. A few participants were concerned about unwilling parents and felt that some nurses might be uncomfortable making such a presentation. To make things easier, a moderator or expert was suggested to accompany nurses to referred websites with information on HPV and vaccination and have a short presentation lasting less than 10 minutes. One nurse doubted the effectiveness of such an intervention in improving vaccine coverage, given that parents already receive all this information.

### Results of MI Training of School Nurses

Regarding the 5-hour web-based course, 24 nurses filled out a pretest and posttest measuring various skills and knowledge. The average rating on the pretest was 66% (n=26) and 85% (n=34) for the posttest. Thus, the average rating increases by 19% in the posttest, which corresponds to an increase of 8 correct answers on average out of a total of 40 questions.

In total, 29 nurses (25 working in schools and 4 working in public health units) attended 1 of the 3 face-to-face training sessions organized at the end of August 2019. All 29 participants completed an evaluation of the training. Overall, they felt the training achieved its objectives (Table 3). The participants found the training and associated exercises interesting, enriching, and relevant.

**Table 3:** Evaluation of the motivational interview training objectives by the school nurses, August 2019.

Training objectives	School nurse evaluation, n (%)				
	1 (totally disagree)	2	3	4	5 (totally agree)
Differentiating MI^a^ from other counseling approaches	—^b^	—	3 (10)	9 (31)	17 (59)
Using MI techniques	—	—	2 (7)	14 (48)	13 (45)
Analyze your MI practice	—	—	1 (3)	12 (41)	16 (55)
Use MI’s know-how	—	—	3 (10)	12 (41)	14 (48)
Using MI tools	—	—	3 (10)	17 (59)	9 (31)
Take a critical look at your MI practice	—	—	1 (3)	12 (41)	16 (55)

^a^MI: motivational interviewing.

^b^Not available.

An integration workshop followed, and nurses were asked to complete a self-assessment grid of what they mastered well in their MI practice and what they would like to improve in a future intervention. After listening to their recording again, she had to assess how much they had applied MI skills (eg, mirroring, valuing, and engaging) in conversation with a simulated hesitant parent.

A total of 25 nurses attended the workshop. According to the MI trainer who led the workshop, 20 nurses were motivated and interested in learning MI, applying it, and improving, and 3 nurses felt moderately comfortable and interested in the training and the project. Finally, 2 other nurses said they were uninterested and did not want to get involved in the training or the project. This was mainly due to their heavy workload, which meant they had little time or interest in participating. This workshop enabled the nurses to assess themselves and receive feedback from the trainer on what they had learned and still needed to improve.

### Results of Patient Decision Aid Formative Development

Following the team’s identification of an unmet need regarding lack of instruction regarding the required provincial consent or refusal form for school-based vaccines, a graduate student involved in the project created a 3-minute animated video explaining to parents how to complete the required paperwork, whether or not they chose to have their child vaccinated.

In the initial formative evaluation with parents and nurses, parents generally appreciated the tool and the information presented. Some said they appreciated the information about transmission and the nuance that the vaccine does not protect against everything. The detailed, research-backed information about vaccine safety and the clickable links were also appreciated. Suggestions were made for additions, such as clarifying specific terms (eg, respiratory papillomatosis) and discussing the prevention of different cancers in boys. Providing exact percentages and simplifying the information with graphics were suggested. Regarding the sliders used to help parents clarify how their values align with their options, all parents in the first group agreed to remove it: “I find it almost insulting: as if you had to choose between side effects and cancer.” Since parents were unanimous on the slider interface, it was not represented in the second group.

In general, the tool and the clarity of the information were appreciated by nurses. It was emphasized that the sources and citations of studies were reassuring and desirable. Suggestions included mentioning free vaccines, adding statistics, and improving the visuals to make them more user-friendly. A total of 2 people also had reservations about the appropriateness of leaving the decision balance in the tool.

In subsequent evaluations, additional concerns were raised about the large quantity of information and data sources. While some parents might desire large amounts of information, it was deemed important not to overwhelm them with all the details. Parents of boys indicated uncertainty about whether the vaccine would be offered to their children. Continued evaluation of the sliders indicated that further information would help users understand its purpose and that it could help parents make values-congruent decisions.

Several changes were made between the first draft by the Université Laval team and the final version. More information on cancer rates, transmission, progression, and screening was added. Information on vaccine safety, ingredients, and side effects was added. To reassure parents, data on efficacy, including in Canada and Quebec, were also included, as well as studies demonstrating that adolescents vaccinated against HPV do not engage in sexual activities earlier than adolescents not vaccinated against HPV. To balance the need for sufficient detail without overwhelming parents who do not want further detail, information was organized to be presented in layers so that users can click to receive more detail if more detail is desired. As for the sliders, the 2 choices were made more nuanced in the final version to better represent how parents conceptualize the decision (Multimedia Appendix 2), similar to the approach used in a previous study about influenza vaccines for children [46].

The final version included the following topics: HPV and cancer, HPV treatment, how to protect your child, the vaccination schedule, vaccine safety, efficacy, and the balance of advantages and disadvantages of vaccination (Multimedia Appendix 3). Clicking on each topic expands the section below, and users can click to see resources and references within each section.

The complementary video was shortened from 7 to 3 minutes. Digital voices were abandoned in favor of human-recorded voices, and greater visible diversity was added to the cartoon characters.

## Discussion

### Principal Findings

This paper presents the results of the intervention development process and formative evaluation. We described the development and formative evaluation of a multifaceted intervention to improve HPV vaccine acceptance and uptake among grade 4 students targeted by the school-based vaccination program in Quebec, Canada.

The formative evaluation was conducted to refine the intervention before the implementation.

Following feedback from the focus group discussions, the presentation content and visuals were improved, and the information was simplified. The results of the MI training for school nurses demonstrated an increase in the skills and knowledge of nurses in using this technique. Several changes were made between the first draft of the web-based decision-aid tool and the final version following an iterative process. More information on cancer rates, transmission, progression, screening, vaccine safety, ingredients, and side effects were added. Finally, the decision-balance slider was more nuanced in the final version.

The results of focus group discussions with school nurses indicated that they appreciated the content, but they shared concerns about the feasibility of such an intervention in the classroom (eg, lack of parent participation, fear of antivaccine parents’ reactions, and presentation being too long). Results of focus groups with parents showed that parents wanted more information on diseases caused by HPV, HPV vaccine safety (eg, possible side effects, vaccine’s ingredients, and safety monitoring) and efficacy, reasons to vaccinate boys, age of administration, and how to prepare their child for the vaccination day. Parents’ concerns and questions were similar to those identified in other studies [8,29,47,48]. In line with these results, the content was reduced and simplified while covering all topics important for parents. However, we did not find published data on the effectiveness of such an in-person intervention to improve the HPV vaccine. Knowledge about the importance of HPV immunization, such an understanding, was underlined as a motivating factor in parents deciding to vaccinate their children [49,50]. A systematic review assessed the effects of face‐to‐face interventions for informing or educating parents about early childhood vaccination [51] and concluded that face‐to‐face interventions may be more effective in populations where a lack of awareness or understanding of vaccination is identified as a barrier. The in-person intervention developed will be an opportunity for parents to learn more about HPV and vaccination, and also, regarding acceptance in school-based programs, 2 systematic reviews indicate that health care provider recommendation of the HPV vaccine is positively associated with vaccination outcomes and completion [52,53]. In the control group, parents will receive, from their school, the consent form and a detailed brochure about the offered vaccine written by the Ministère de la Santé et des services sociaux du Québec [45].

The evaluation of the nurses’ training for the phone call reminder using MI techniques showed increased skills and knowledge. Most nurses were motivated and interested in learning and applying MI at the workshop. MI is a brief intervention style based on empathic listening, absence of argumentation, and respect for autonomy that is used to help an individual make an informed decision and reinforce their motivation and commitment to health-promoting behavior [36]. The success of MI training and the literature suggest that this intervention can potentially positively impact HPV vaccination acceptance [54,55]. MI has been highly effective in enhancing vaccine coverage for routine childhood vaccines in Quebec [54,56]. This technique also demonstrated efficacy in decreasing parents’ vaccine hesitancy [54]. Other studies conducted in the United States have also indicated a positive impact on HPV vaccine acceptance, albeit not in school-based programs [27]. In the United States, a research team implemented an intervention to improve provider communication with HPV vaccine-hesitant parents by offering communication training, including MI techniques [57], and assessed the efficacy of this intervention. Their findings indicated that “the intervention improved providers’ communication with HPV vaccine-hesitant parents, and the use of MI played a central role in improved HPV vaccine acceptance” [57]. In addition, a study indicates that health care provider recommendation is a primary reason for patient uptake of the HPV vaccine and that educational interventions should also target nurses as they are key informants about vaccine-related information [58]. In the control group, the school nurses carried out their activities as usual; it is possible that some nurses made calls to parents who had not returned the consent form. Nevertheless, these nurses will not have received the MI training that was offered to those in the pilot schools. However, it is possible that some of these nurses have already had MI training in the course of their careers.

Prospective users generally appreciated the content and references provided regarding the patient decision aid. A few suggestions were made to round out the information, improve its visual appeal, and make it more accessible to a broader audience. Through iterative refinement and collaboration between experts in web design and development, decision science, public health, and immunization, we aimed to ensure that the patient decision aid and accompanying video met the standards set by public health experts while remaining useful and relevant to parents and guardians making decisions about HPV vaccination for their children. The patient decision aid is positioned as part of a broader suite of interventions because not everyone feels uncertainty about vaccine decisions or desires more information. Even within the group of people who want more information, some want a little, while others want a lot. Through iteration, we settled on an approach of layering information, meaning that people were automatically offered brief information, but they could click for more details and click again to see references. Layering information in this way allows people to tailor information to their needs and preferences, a longstanding advantage of providing information via the web rather than in a one-size-fits-all verbal or printed presentation [59]. A systematic review and meta-analysis concluded that patient decision aids could assist in vaccine decision-making [35]. In the control group, nurses sent a reminder to parents who had not returned their signed consent form, most often by a note sent by the school or a message in the student’s diary. Based on the literature, a reminder with a decision aid tool may be helpful for undecided parents [35].

A French study shared information about the development of a decision aid tool as part of a multicomponent intervention [60] to support hesitant parents or adolescents by providing information on options and associated advantages and disadvantages and helping to clarify the fit between decisions and personal values. In their process, they also involved the public in the development process to revise the tool and provide advice about the intervention practicalities, feasibility, and maintenance. Like us, they involved regional and national stakeholders as a strength of the participatory approach. Authors underlined this approach in the development of new interventions to have a better chance of being effective when evaluated and then adopted on a larger scale in the real world [60].

The development process has several strengths as the interventions were based on published research evidence and results from an evaluation phase on target populations’ needs to address barriers to HPV vaccination. A participatory approach in a coconstruction process involving many parents, nurses, experts, and regional and national stakeholders (eg, Ministry of Health and Social Service and immunization program managers) has been conducted. The opinions of nurses and stakeholders about the feasibility of the implementation were considered for further steps.

### Limitations

The main limitation of our research was the need to develop interventions within a timeline that prevented data collection from larger groups of people. While focus groups have their advantages (eg, multiple perspectives and flexibility), this method also has its limitations. Although we had 2 groups of parents and 1 of nurses, the small sample size and specific demographic characteristics of the participants may not accurately represent the population as a whole. It is also possible that some participants did not share their true thoughts and conformed to the group’s opinions. MI is a method that involves a certain complexity. Although nurse training has covered the theoretical aspects and included exercises and applications, it can remain a difficult skill to acquire for some nurses who have less time or ability to progress in their learning and application, also, we did not collect data on the school nurses in the control schools, which may limit our understanding of the activities they carried out and their knowledge of MI techniques.

Future research within this project will examine the effects of the suite of interventions on a large group of parents and guardians.

### Conclusion

In conclusion, the paper details the development and formative evaluation of interventions to improve HPV vaccine acceptance and uptake among grade 4 students’ parents in Quebec, Canada. Despite the existence of school-based vaccination programs, disparities in vaccine coverage persist, particularly in socially and materially deprived areas and regions with a higher proportion of immigrants. Barriers to vaccine acceptance and uptake include parental attitudes, knowledge, and beliefs, and system-level issues such as the informed consent process.

The paper discusses the development of 3 interventions: an in-person presentation by school nurses, an email reminder with a web-based information and decision aid tool, and a telephone reminder using MI techniques. Interventions were developed and evaluated in collaboration with content experts. Based on feedback from focus groups with parents and nurses, the interventions were refined, addressing concerns and optimizing the content for better understanding and acceptance.

The results of the MI training for school nurses demonstrated an increase in skills and knowledge, indicating a positive impact on HPV vaccine acceptance. The development of a patient decision aid, including an animated video, aimed to provide parents with validated information to support informed decision-making. The iterative refinement process involved feedback from parents, nurses, and experts to ensure the tool’s relevance and effectiveness.

Future research will focus on evaluating the effectiveness of these interventions on a larger scale. Overall, this comprehensive approach seeks to enhance vaccine coverage and contribute to the prevention of HPV-related cancers and infections in the target population.

## Appendix

Multimedia Appendix 1 Recruitment questionnaire for focus group participants.

Multimedia Appendix 2 Example of changes in the decision aid tool (intervention 2).

Multimedia Appendix 3 Example of themes in the decision aid tool (intervention 2).

## Abbreviations

CHU: Centre Hospitalier Universitaire
HPV: human papillomavirus
INSPQ: Institut National de Santé Publique du Québec
MI: motivational interviewing
