# Supplementary material for: Development of Promising Interventions to Improve Human Papillomavirus Vaccination in a School-Based Program in Quebec, Canada: Results From a Formative Evaluation Using a Mixed Methods Design
Source: JMIR Form Res. 2024 Jul 8;8:e57118. doi: 10.2196/57118 (PMC11263894; doi:10.2196/57118)
Supplement: Multimedia Appendix 1 [file formative_v8i1e57118_app1.docx]

**RECRUITMENT QUESTIONNAIRE**

**FOCUS GROUPS**

To find out if you have the right profile, complete the qualification questionnaire by clicking on the button below:

1. Do you or a family member work in the following sectors?

- A research company (marketing, surveys)
- An advertising company
- The media (newspapers, TV, radio, magazines)
- Health and social services sector
- Pharmaceutical industry
- None of the above

2. When did you last participate in a paid discussion group?

- Less than three months ago
- Three to six months ago
- Six months to less than a year ago
- One to less than two years ago
- More than two years ago
- I have never participated in a paid discussion group
- I don't know / I prefer not to answer

3. Are you the parent or guardian of a child in grade 3?

- Yes, I have a daughter in grade 3
- Yes, I have a boy in grade 3
- No, I don't have a child in grade 3
- I prefer not to answer

4. How involved are you in decisions affecting the health of your child in 3rd grade?

- I am solely responsible for these decisions
- I am jointly responsible for these decisions
- I am not involved in these decisions
- I prefer not to answer

5. How much do you trust vaccination to protect your child(ren)'s health?

- Totally confident
- Somewhat confident
- Slightly confident
- Not at all confident
- I prefer not to answer

6. When it comes to vaccinating your child(ren) against infectious diseases such as measles, mumps, polio and chickenpox, which of the following statements best describes your approach?

- I have accepted all the vaccines recommended for my child(ren).
- I have accepted some vaccines but postponed or refused others
- I have refused most or all of the vaccines recommended for my child(ren).
- I prefer not to answer

7. What age group do you belong to?

- 18 to 24 years
- 25 to 34 years
- 35 to 44 years old
- 45 to 54 years old
- 55 and over
- *I prefer not to answer

8. What is the highest level of education you have completed?

- High school or less
- College not completed
- College completed
- University not completed
- University degree obtained (Bachelor's, Master's, Doctorate)
- *I prefer not to answer
