# Supplementary figures and images for: Development of Promising Interventions to Improve Human Papillomavirus Vaccination in a School-Based Program in Quebec, Canada: Results From a Formative Evaluation Using a Mixed Methods Design
Source: JMIR Form Res. 2024 Jul 8;8:e57118. doi: 10.2196/57118 (PMC11263894; doi:10.2196/57118)

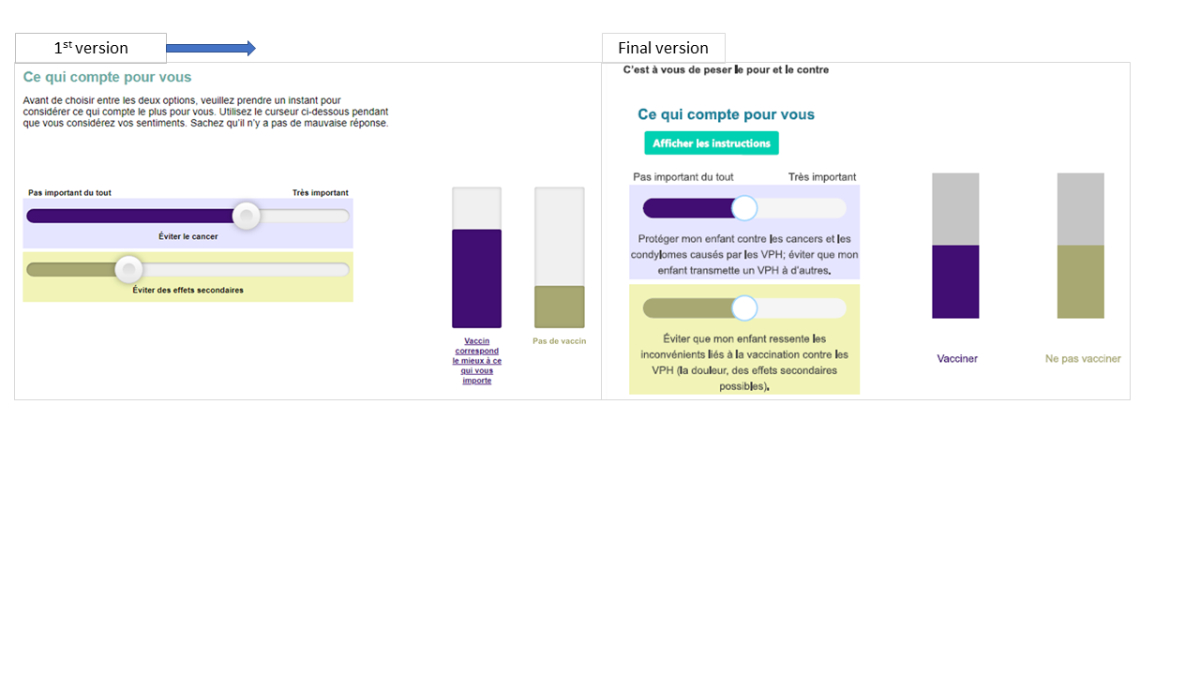

Supplement: Multimedia Appendix 2 [file formative_v8i1e57118_app2.png]

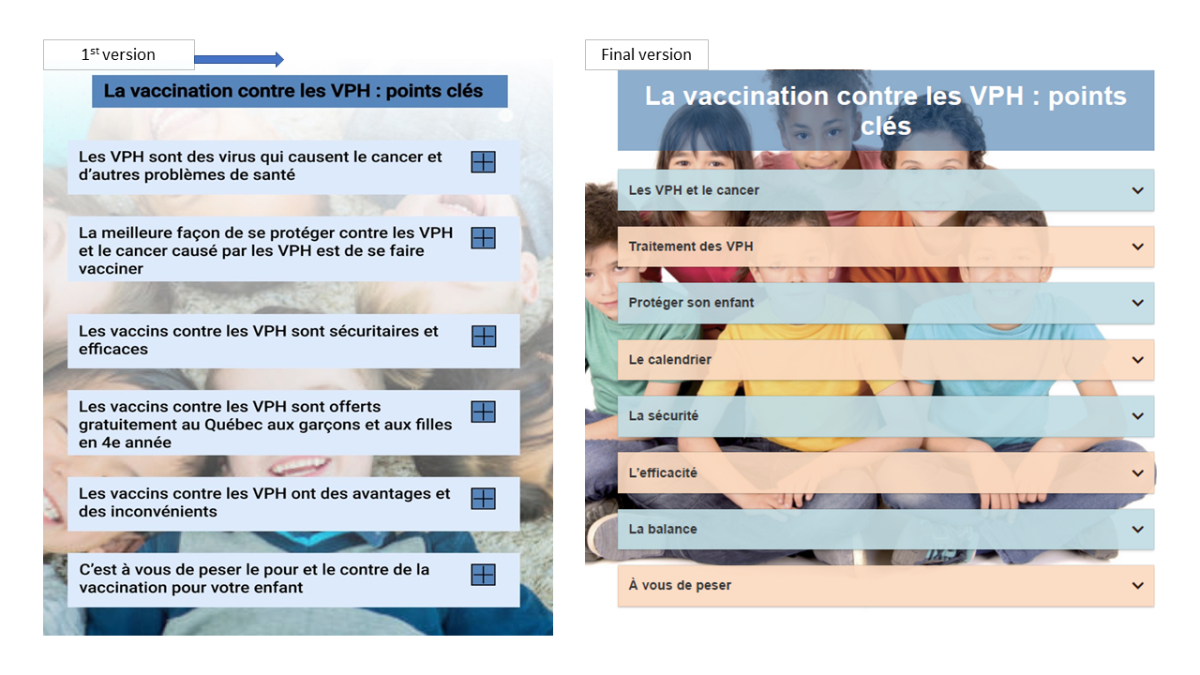

Supplement: Multimedia Appendix 3 [file formative_v8i1e57118_app3.png]
